# Transcriptomic Analysis Revealed the Response Mechanism of Green Revolution Gene *Rht-D1b* in Wheat to Salt Stress

**DOI:** 10.3390/life16101632

**Published:** 2026-09-29

**Authors:** Jinlan Ni, Fulin Yang, Weixiang Wang, Ziyi Chen, Xiaomin Guo, Yuanfeng Hao, Dengan Xu, Wujun Ma, Mei Qu

**Affiliations:** 1 College of Agronomy, Qingdao Agricultural University, Qingdao 266109, China; 2 National Center of Technology Innovation for Comprehensive Utilization of Saline-Alkali Land, Dongying 257347, China; 3 Institute of Crop Sciences, Chinese Academy of Agricultural Sciences (CAAS), Beijing 100081, China; 4 Open Research Projects of the State Key Laboratory of Wheat Breeding, Shandong Academy of Agricultural Sciences, Jinan 250100, China; 5 Australia-China Joint Centre for Wheat Improvement, State Agriculture Biotechnology Centre, Murdoch University, Perth, WA 6150, Australia; 6 Academy of Dongying Efficient Agricultural Technology and Industry on Saline and Alkaline Land in Collaboration with Qingdao Agricultural University, Dongying 257347, China

**Keywords:** wheat, salt stress, physiological mechanism, transcriptome

## Abstract

The semi-dwarfing gene *Rht-D1b* (formerly known as *Rht2*) was introduced into modern wheat breeding during the Green Revolution to reduce plant height, improve lodging resistance, and increase harvest index. In this study, two wheat (*Triticum aestivum* L.) lines carrying *Rht-D1b* (dwarf) and *Rht-D1a* (tall), respectively, were used to assess the role of *Rht-D1b* in salt tolerance through physiological and transcriptomic analyses under salt stress. Compared with *Rht-D1a*, *Rht-D1b* showed less growth inhibition, with smaller decreases in plant height, root length, and shoot biomass, as well as higher SPAD values. *Rht-D1b* also accumulated less Na^+^, had lower ROS and MDA levels, and maintained stronger POD and CAT antioxidant enzyme activities. Transcriptome analysis showed that 20 POD genes were consistently upregulated in *Rht-D1b* to *Rht-D1a* at 0, 6, and 24 h, while ion-transport- and ROS-related genes showed time-specific activation patterns linked to Na^+^ homeostasis, calcium signaling, and antioxidant defense. Together, these findings suggest that *Rht-D1b* may contribute to wheat seedling salt tolerance by improving ion homeostasis and redox balance. The salt-responsive genes identified in this study may also serve as candidates for future functional studies and wheat breeding.

## 1. Introduction

Soil salinization is one of the main abiotic stress factors limiting global agricultural productivity. It poses a serious threat to crop growth, development, yield, and quality [1]. According to statistical data, more than 20% of the world’s irrigated farmland has already been affected by salinization. Driven by climate change, unsustainable irrigation practices, and sea-level rise, this proportion continues to increase [2]. Wheat (*Triticum aestivum* L.) is one of the most important staple food crops globally, providing approximately 20% of the protein and caloric intake for humans. However, wheat is classified as a moderately salt-tolerant crop. High-salinity environments can cause significant yield reductions or even complete crop failure [3]. The effects of salt stress on plants can be divided into three main phases. First, “osmotic stress” is caused by a decrease in osmotic potential around the roots, which hinders water uptake. Second, as salt ions (mainly Na^+^) accumulate in plant tissues, they trigger “ion toxicity”. Over time, this ultimately leads to nutrient imbalance and oxidative stress damage [4]. Under salt-stressed conditions, wheat exhibits inhibited seed germination, stunted growth, leaf scorching, and abnormal spike development, ultimately limiting its suitable geographic range and production potential [5]. Therefore, it is strategically important to explore genetic resources for salt tolerance in wheat and to elucidate the underlying physiological and molecular mechanisms, which are essential for breeding new salt-tolerant wheat varieties and ensuring global food security [6,7].

During long-term evolution, plants have evolved complex regulatory mechanisms to cope with salt stress [8]. When subjected to high salt stress, plants maintain their survival through the coordinated adjustment of morphological, physiological, and biochemical characteristics. First, to resist osmotic stress, plants accumulate large amounts of substances for osmotic regulation in the cytoplasm. These substances include carbohydrates (such as sucrose and fructose), amino acids (especially proline), and quaternary ammonium compounds (such as betaine) [9]. Under salt stress, maintaining ionic homeostasis is crucial for plant salt tolerance. Salt-tolerant plants limit excessive Na^+^ accumulation and enhance the uptake of essential cations such as K^+^, Mg^2+^, and Ca^2+^. This helps restore ion homeostasis and alleviate salt-induced damage [10]. These physiological responses depend on transcriptional activation or repression of downstream genes. Transcriptional regulation is a critical step [11]. Among the many stress-responsive regulators, DELLA proteins are key negative regulators of gibberellin signaling. They have been reported to play roles in various abiotic stresses, such as salt stress [12].

During the “Green Revolution” of the 1960s, the introduction of the reduced-height (*Rht*) gene completely transformed the plant architecture of wheat, significantly improving its harvest index and resistance to tillering [13]. Among these genes, *Rht-B1b* and *Rht-D1b* (formerly known as *Rht1* and *Rht2*) are the two most widely used alleles in current agricultural production [14]. They encode DELLA proteins, which act as negative regulators in the gibberellin (GA) signal transduction pathway [15]. *Rht-D1a* (wild type) encodes a functional DELLA protein that is degraded upon GA induction, thereby promoting plant elongation, whereas *Rht-D1b* (the mutant allele) encodes a GA-insensitive truncated protein, leading to its excessive accumulation within cells, which inhibits cell elongation and ultimately results in a dwarf phenotype [16]. Although *Rht-D1b* has made significant contributions to yield enhancement, its response to abiotic stresses such as salt stress and drought remains controversial [17,18]. Some studies suggest that the growth inhibition caused by dwarfing genes may confer better stress tolerance, as reduced leaf area and limited growth rate help decrease water consumption and ion accumulation. Other studies indicate that *Rht-D1b* may reduce biomass accumulation and compensatory growth capacity under stress conditions [19]. Recent research has uncovered a novel mechanism by which Green Revolution genes participate in abiotic stress responses. The post-translational modification of DELLA proteins, especially SUMOylation, can enhance salt tolerance in rice by modulating the interaction between DELLA proteins and downstream transcription factors [20]. This finding provides a new molecular perspective for dissecting the impact of *Rht-D1* alleles on wheat salt tolerance.

Plant salt tolerance largely depends on ion transport and ROS homeostasis. The *HKT*, *NHX*, and *SOS* gene families are key regulators of Na^+^ transport, sequestration, and efflux [21,22]. HKT proteins contribute to Na^+^ and K^+^ transport, and wheat *HKT1* has been reported to function as a Na^+^/K^+^ symporter [23]. The *NHX* family mediates vacuolar Na^+^ sequestration and helps reduce cytoplasmic Na^+^ toxicity [24,25]. This role is supported by studies showing that ectopic expression of vacuolar *NHX* genes from rice and *A. gmelinii* improves salt tolerance in transgenic plants [26,27]. The *SOS* pathway, which includes *SOS1*, *SOS2*, and *SOS3/SCaBP8*, regulates Na^+^ efflux across the plasma membrane [28,29]. In addition, *SOS2* can promote K^+^ influx through the *SCaBP8–AKT1* module and can also regulate light-dependent salt tolerance through *PIF1/PIF3* degradation [30]. Beyond ion transport, Ca^2+^-ROS signaling is also important for salt-stress responses. *CNGC* genes participate in cytosolic Ca^2+^ signaling and early salt perception, although different members may have different effects on salt tolerance [31,32,33]. *Rboh* genes are major sources of salt-induced ROS production. Salt-triggered increases in cytosolic Ca^2+^ can activate Rboh proteins and promote apoplastic H_2_O_2_ accumulation [34,35]. Excess ROS is then controlled by antioxidant enzymes, including SOD, CAT, and POD, which help maintain cellular redox balance [36,37,38]. In wheat, salt-responsive expression of *TaSOD1.7* has been reported [39]. The *TaPDI-15/TaCRT1-1* complex and *TaPRX-2A* also enhance salt tolerance by maintaining antioxidant enzyme activities and promoting ROS clearance [40]. Together, these studies indicate that ion-transport-, Ca^2+^-ROS-signaling-, and antioxidant-related gene families are useful candidates for studying wheat salt tolerance. However, their expression responses in the *Rht-D1b* and *Rht-D1a* lines remain unclear. Therefore, this study examined the differential expression of these gene families to explore the molecular basis of *Rht-D1b*-associated salt tolerance.

Near-isogenic lines (NILs) serve as ideal materials for studying the function of individual genes. However, NILs developed through repeated backcrossing require multiple generations and are not always available for a given gene of interest [41]. Spontaneous mutants with high genome-wide similarity to their parental lines can serve as alternative materials for allele function analysis [42]. By comparing the phenotypic expressions of *Rht-D1a* and *Rht-D1b* in the same genetic background, the regulatory role of this gene locus in wheat salt tolerance can be precisely elucidated. However, research on the transcriptional regulatory networks and metabolic profiles of different *Rht-D1* alleles under salt stress remains limited, constraining our in-depth understanding of the pleiotropy of “Green Revolution” genes under abiotic stress conditions.

Although extensive research has been conducted on wheat salt tolerance, comparative studies of the two *Rht-D1* lines under salt stress are remarkably limited. In particular, the salt-induced transcriptional reprogramming differences between *Rht-D1a* and *Rht-D1b* are poorly characterized. Systematic, large-scale data on how these differences contribute to stress responses are still lacking. In this study, wheat lines carrying *Rht-D1a* and *Rht-D1b* were subjected to salt stress. High-throughput RNA-seq was employed to comprehensively analyze the dynamic response patterns of the two genotypes at the transcriptomic level. The core objectives of this study are as follows: (1) to determine whether the *Rht-D1b* dwarfing gene alters the sensitivity and tolerance of wheat to salt stress; (2) to identify core differentially expressed genes regulated by the *Rht-D1* locus; (3) to construct an *Rht-D1*-mediated regulatory network of salt stress responses, and to elucidate the molecular mechanisms by which this pathway regulates osmotic balance, ion homeostasis, and hormone signaling under salt stress. The results of this study will enrich the theoretical framework of wheat salt tolerance. They will also provide new evidence for the stress biology functions of Green Revolution genes. Furthermore, they will offer important candidate gene resources and theoretical guidance for breeding new high-yielding, salt-tolerant wheat varieties through molecular design breeding.

## 2. Materials and Methods

### 2.1. Plant Materials and Growth Conditions

The *Rht-D1b* mutant (Zhongyou 9507s) arose as a spontaneous mutant from the *Rht-D1a* cultivar Zhongyou 9507, and seeds of both lines were obtained from the National Wheat Improvement Center, Chinese Academy of Agricultural Sciences. We used Sanger sequencing to genotype the *Rht-D1* locus, which confirmed that the two materials carry the *Rht-D1a* and *Rht-D1b* alleles, respectively. 50K SNP chip genotyping further showed that the two lines share over 89% genome-wide genetic similarity, with 59,693 consistent SNPs out of a total of 66,835 detected SNPs (Table S1). These lines are not strict near-isogenic lines, and residual background differences may still exist. This experiment included two treatments, namely control conditions (0 mM NaCl) and salinity stress (200 mM NaCl). One hundred seeds were sterilized for 20 min using a 1:1 diluted 3% hydrogen peroxide, followed by at least five washings with deionized distilled water [43]. The sterilized seeds were placed in Petri dishes containing filter paper moistened with distilled water and incubated in complete darkness at room temperature (25 ± 2 °C) for 2 days. After germination, seedlings of similar size were selected and transferred to hydroponic culture containing 1/5 Hoagland nutrient solution (pH 5.8) and continuously aerated with an air pump [44]. After 4 days of normal growth in Hoagland nutrient solution, the control group was refreshed with new 1/5 Hoagland nutrient solution (pH 5.8), while the salt group was switched to 100 mmol/L NaCl and then changed to 200 mmol/L NaCl the following day. Plants were cultivated under white fluorescent light (18/6 h light-dark cycle) at a temperature of 20 °C, relative humidity of 50–55%, and light intensity of 150 μmol m^−2^ s^−1^ [45]. The measurement of relevant indicators is performed after 7 days of treatment. The above experiment was conducted in three biological replicates.

### 2.2. Growth and Morphological Measures

The 7-day-old seedlings of two genotypes were observed for differences and recorded by taking photos with a camera (Canon 80D). For each wheat plant, SPAD value was measured on leaves using a handheld chlorophyll meter (SPAD-502; Konica Minolta, Osaka, Japan), with three readings taken per leaf and then averaged [46]. Root length and shoot length were measured (cm) using a one-meter ruler. Fresh weights of roots and shoots were obtained using an analytical balance (g). Then the fresh samples were oven-dried at 65 °C for 120 h to a constant weight, and the root dry weight and shoot dry weight were determined [47].

### 2.3. Determination of Potassium and Sodium Ions

Potassium and sodium ions were determined using a M410 (Sherwood-2020, Sherwood Scientific Ltd., Cambridge, UK) flame photometer. A 0.2 g dried sample was ground, then 10 mL of distilled water was added, followed by extraction in a boiling water bath for 2 h. After the extract was filtered and diluted, the Na^+^ and K^+^ concentrations in the filtrate were determined using a flame photometer [48].

### 2.4. Determination of ROS, Lipid Peroxidation Products, and Antioxidant Enzyme Activities

We measured the levels of reactive oxygen species (ROS; kit GY0163W), malondialdehyde (MDA; kit G0110W), superoxide dismutase (SOD; kit G0102W), peroxidase (POD; kit G0108W), and catalase (CAT; kit G0106W) using commercial assay kits from Suzhou Grace Biotechnology Co., Ltd. (Suzhou, China), with three biological replicates for each parameter. All procedures were carried out in strict accordance with the manufacturer’s protocols.

### 2.5. Transcriptomic Analysis

The transcriptomic sequencing workflow includes RNA extraction, quality assessment, library construction, and sequencing. Roots were collected at three time points: 0 h (control), 6 h, and 24 h after treatment with 200 mmol/L NaCl for the “*Rht-D1a*” wild-type and “*Rht-D1b*” semi-dwarf mutant wheat. Three biological replicates were set up for each treatment, totaling 18 samples, which were grouped into 2 genotypes × 3 time points × 3 biological replicates. Then the root samples were washed, frozen rapidly in liquid nitrogen, and stored at −80 °C. cDNA libraries were constructed following the standard Illumina protocol. Total RNA was extracted from root tissue, ensuring RNA integrity (RIN) of ≥8.0 following assessment. Dual-end 150 bp (PE150) sequencing was performed by Sangon Biotech Co., Ltd. (Shanghai, China) using the DNBSEQ-T7 platform.

Raw reads were processed with FASTP v0.23.2 to remove adapter sequences, low-quality bases, and short reads. The resulting clean reads were then aligned to the wheat reference genome (IWGSC RefSeq v2.1) using HISAT2 v2.2.1. FeatureCounts v2.0.3 was employed to count the aligned reads per gene, and gene expression levels were calculated using the TPM methods. Differential expression analysis was performed using DESeq2 v1.42.0, and differentially expressed genes (DEGs) were identified based on the criteria of |log_2_FC| ≥ 1 (i.e., fold change ≥ 2 for DEGs) and *P*-adj < 0.05. Low-expressed genes (rowSums of TPM < 2) and low-confidence genes (gene names containing ‘LC’) were filtered out during the analysis. Through pairwise comparisons between groups, differentially expressed genes (DEGs) were identified at each of the three time points. Functional annotation and pathway enrichment analysis (*p* < 0.05) were performed using the KEGG database (https://www.kegg.jp/) URL (accessed on 23 July 2026 ).

### 2.6. Identification of Salt-Tolerance-Related Gene Family Members

Eight gene families associated with salt tolerance were analyzed in this study, including catalase (*CAT*), Na^+^/H^+^ exchanger (*NHX*), superoxide dismutase (*SOD*), cyclic nucleotide-gated channel (*CNGC*), high-affinity potassium transporter (*HKT*), respiratory burst oxidase homolog (*Rboh*), class III peroxidase (*POD*), and salt overly sensitive (*SOS*) gene families. Members of the *CAT*, *NHX*, *SOD*, *CNGC*, *HKT*, and *Rboh* gene families in wheat have been systematically identified and named at the genome-wide level in previous studies [22,49,50,51,52]. For the *POD* and *SOS* gene families, however, no such genome-wide identification has been performed. A previous study only examined the stress-responsive expression of SOS-related genes and did not attempt a genome-wide identification of the SOS family in wheat [48]. In the present study, we used homology-based searches to identify their members in wheat. The previously identified class III POD protein sequences from grape (*Vitis vinifera* L.) and SOS protein sequences from tomato (*Solanum lycopersicum* L.) were collected as query sequences [53,54].

BLASTP searches were performed against the protein database of the wheat reference genome IWGSC RefSeq v2.1, with an *E*-value < 1e − 5. Candidate sequences were further examined using HMMER v3.0, together with the Pfam and SMART databases, to confirm the presence of conserved domains. Only sequences containing complete and corresponding conserved domains were retained. These domains included the plant peroxidase domain PF00141 for POD proteins and characteristic domains of SOS-related proteins, such as Na_H_Exchanger, PKc_like, and FRQ1. After domain confirmation, the retained sequences were defined as members of the wheat *TaPOD* and *TaSOS* gene families.

### 2.7. Statistical Analysis

The experiment was conducted as a two-factor factorial arrangement with three replicates, laid out in a completely randomized design. All data were collated and organized using Excel 2019. Statistical analysis was performed using IBM SPSS Statistics 19. A two-way analysis of variance (two-way ANOVA) was performed to examine the differences between the two varieties under salt stress. Mean separation was conducted using Duncan’s multiple range test. Different letters indicate significant differences at *p* < 0.05 [55]. Data visualization was carried out using GraphPad Prism 10.6.0.

## 3. Results

### 3.1. Analysis of Phenotypic Differences Between Rht-D1a and Rht-D1b After Salt Stress

**Figure 1:**
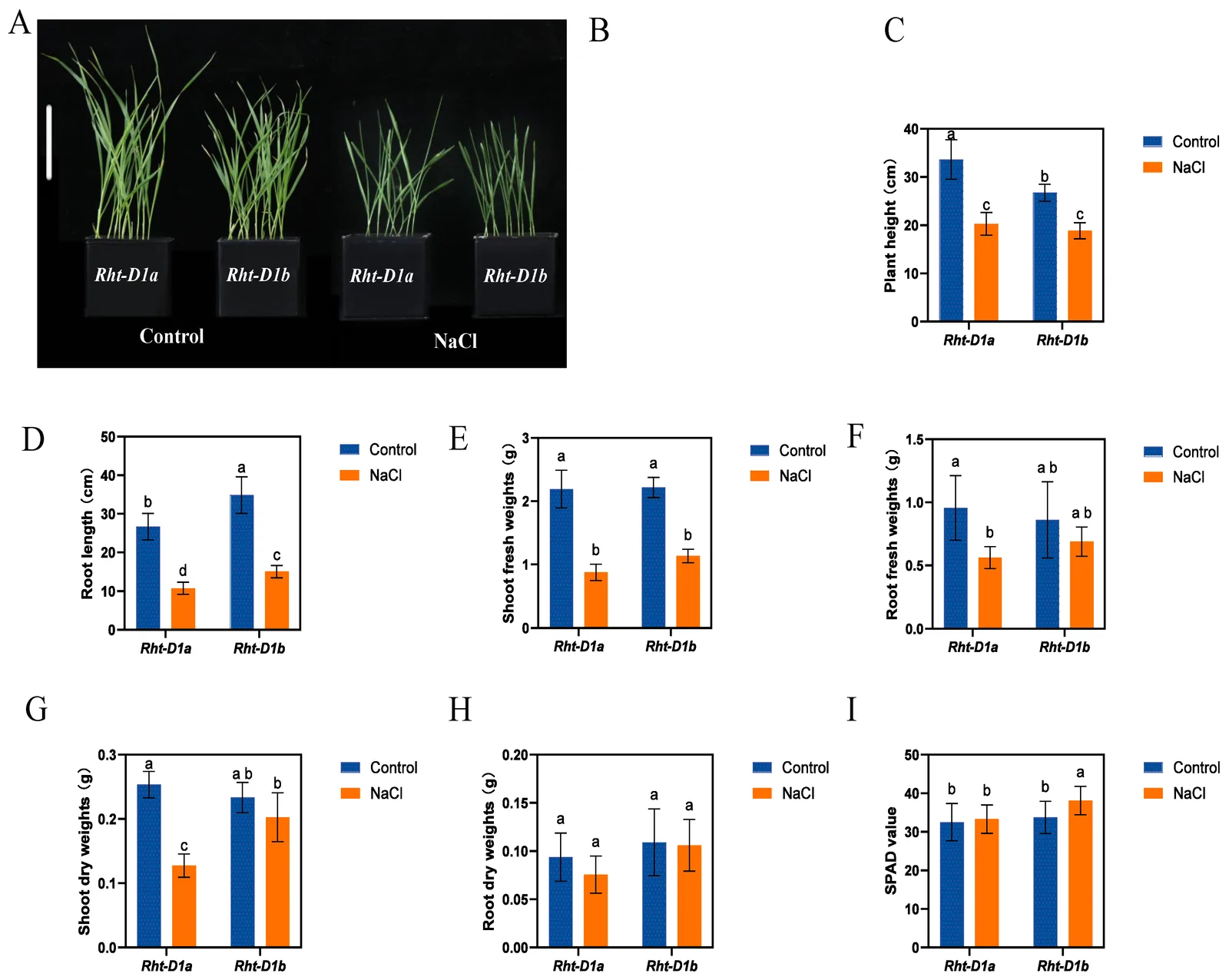
Effects of 200 mM salt stress on the phenotype, growth parameters, and chlorophyll content (SPAD) of wheat *Rht-D1a* and *Rht-D1b* lines. (**A**) Whole plant phenotypes of *Rht-D1a* and *Rht-D1b* wheat lines under control and 200 mmol/L NaCl salt stress conditions. Scale bar = 10 cm; (**B**) seedling shoot and root morphology of the two wheat lines under control and salt treatment. Scale bar = 5 cm; (**C**) plant height; (**D**) root length; (**E**) shoot fresh weight. *n* = 5; (**F**) root fresh weight. *n* = 5; (**G**) shoot dry weight. *n* = 5; (**H**) root dry weight. *n* = 5. (**I**) SPAD values. The different letters represent significant differences according to Duncan’s multiple range test, *p* < 0.05.

Wheat carrying the semi-dwarfing allele of *Rht-D1b* exhibits excellent lodging resistance, enabling a significant increase in wheat yield per unit area [56]. Based on this foundation, we further explored the differences in response to salt stress between the *Rht-D1b* allele and the wild-type *Rht-D1a* allele. The results indicate that the growth of both wheat genotypes was significantly inhibited under 200 mmol NaCl conditions (Figure 1A,B). Compared with the untreated control, the plant height of *Rht-D1a* decreased by 39.63%, while that of *Rht-D1b* decreased by 29.53% (Figure 1C). The magnitude of plant height reduction was significantly greater in *Rht-D1a* than in *Rht-D1b*, indicating that under salt stress conditions, the degree of shoot growth inhibition in *Rht-D1b* was weaker than that in *Rht-D1a.* Under salt stress, the root length of *Rht-D1a* and *Rht-D1b* decreased significantly, showing reductions of 59.80% and 56.94%, respectively, compared with the control (Figure 1D). Under control conditions, there was no significant difference in shoot fresh weight between the *Rht-D1a* and *Rht-D1b* lines; under salt stress treatment, shoot fresh weight decreased significantly in both lines, by 60.05% and 48.83%, respectively (Figure 1E). Salt stress induced a significant 42.56% reduction in root fresh weight of the *Rht-D1a* genotype, whereas there was no significant difference in root fresh weight of *Rht-D1b* compared with the control group (Figure 1F). Accordingly, we can draw a preliminary conclusion that under salt stress conditions, *Rht-D1b* presents a significantly better growth phenotype than *Rht-D1a*. The shoot dry weight of *Rht-D1a* decreased significantly by 49.75% under salt stress (Figure 1G), while that of *Rht-D1b* only exhibited a slight, non-significant decrease. Changes in root dry weight followed a similar pattern to that of root fresh weight; we therefore proceeded to measure the photosynthetic characteristics of these two wheat materials. Salt stress inhibits photosynthesis, and a significant positive correlation has been confirmed between chlorophyll content and SPAD value [57]. Under salt stress, the SPAD value of the *Rht-D1b* genotype increased significantly (Figure 1I), which can likely be attributed to enhanced photosynthesis in the tested newly emerged leaves, as a protective response against salt stress.

### 3.2. Analysis of Physiological and Biochemical Differences Between Rht-D1a and Rht-D1b After Salt Stress

**Figure 2:**
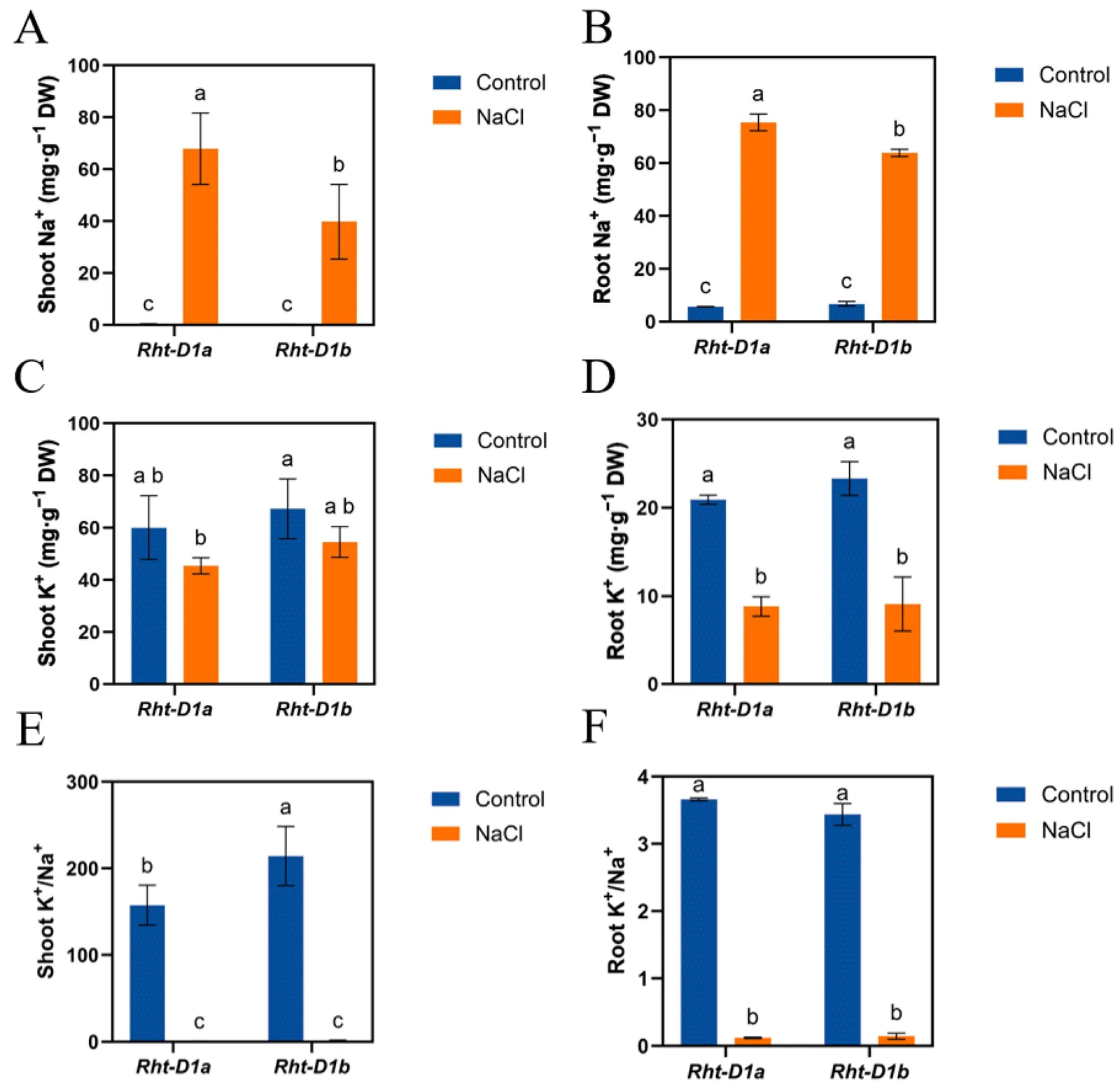
Effects of 200 mM salt stress on sodium, potassium ion content, and potassium–sodium ion ratio of wheat *Rht-D1a* and *Rht-D1b* lines. (**A**) Effect of 200 mmol/L salinity on shoot sodium (Na^+^) content of *Rht-D1a* and *Rht-D1b* wheat lines; (**B**) effect of 200 mmol/L salinity on root Na^+^ content of the two wheat lines; (**C**) effect of 200 mmol/L salinity on shoot potassium (K^+^) content of the two wheat lines; (**D**) effect of 200 mmol/L salinity on root K^+^ content of the two wheat lines; (**E**) K^+^/Na^+^ ratio on shoot; (**F**) K^+^/Na^+^ ratio on root. Different letters represent significant differences according to Duncan’s multiple range test, *p* < 0.05.

The adverse effects of salt stress are closely associated with excessive Na^+^ accumulation in plants, and maintaining a stable K^+^/Na^+^ ratio in the cytoplasm is a core characteristic of plant salt tolerance. [58]. We measured the ion concentrations in the plant shoots and roots to clarify the differences in Na^+^ and K^+^ accumulation between these different tissues. The results indicate that under salt stress, Na^+^ content increased significantly in the shoots of both *Rht-D1a* and *Rht-D1b*, whereas *Rht-D1b* accumulated significantly less Na^+^ than *Rht-D1a* (Figure 2A). Although the K^+^ content did show a decrease, no significant difference was observed between the two tested wheat lines (Figure 2C). The K^+^/Na^+^ ratios for both *Rht-D1a* and *Rht-D1b* decreased significantly (Figure 2E), suggesting that *Rht-D1b* may exhibit relatively better salt tolerance by maintaining lower Na^+^ accumulation. In both *Rht-D1a* and *Rht-D1b* genotypes, root Na^+^ content increased significantly (Figure 2B), but the increase was greater in *Rht-D1a*. Both K^+^ levels showed a significant decrease (Figure 2D), and the K^+^/Na^+^ ratios for both *Rht-D1a* and *Rht-D1b* decreased significantly (Figure 2F). This indicates that the salt tolerance mechanisms in the roots are consistent with those in the aboveground parts and that *Rht-D1b* exhibits greater salt tolerance.

**Figure 3:**
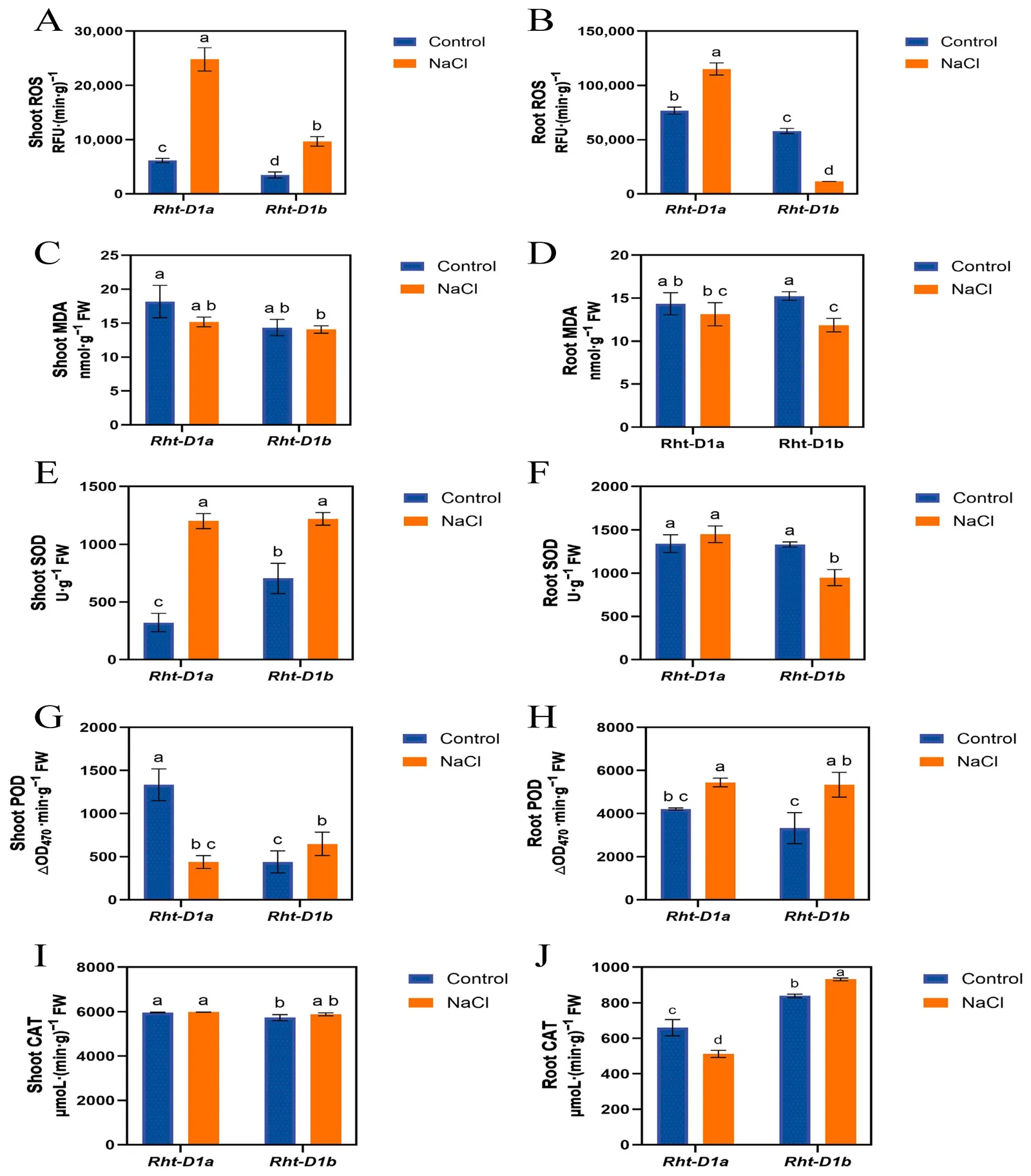
Effects of 200 mM salt stress on oxidative damage indicators and antioxidant enzymes of wheat *Rht-D1a* and *Rht-D1b* wheat lines. (**A**) Reactive oxygen species (ROS) content on shoot; (**B**) ROS content on root; (**C**) malondialdehyde (MDA) content on shoot; (**D**) MDA content on root; (**E**) superoxide dismutase (SOD) activity on shoot; (**F**) SOD activity on root; (**G**) peroxidase (POD) activity on shoot; (**H**) POD activity on root; (**I**) catalase (CAT) activity on shoot; (**J**) CAT activity on root. The different letters represent significant differences according to Duncan’s multiple range test, *p* < 0.05.

Salt stress triggers reactive oxygen species (ROS) accumulation in plant cells. While low levels of ROS act as signaling molecules that activate salt stress response pathways, excessive ROS accumulation can exert severe cytotoxic effects on cells [59]. Therefore, effectively maintaining the dynamic balance of ROS through precise regulation is crucial for plants to tolerate and adapt to salt stress. Under salt stress, *Rht-D1a* exhibited a significant increase in ROS accumulation in both shoots and roots (Figure 3A), while *Rht-D1b* showed a significant decrease in ROS accumulation in its roots (Figure 3B). Although shoot ROS levels increased in both genotypes, the total ROS accumulation in *Rht-D1b* was 60.96% lower in shoots and 89.91% lower in roots than that in *Rht-D1a.* Excessive accumulation of ROS within cells can disrupt cellular membrane systems, triggering a significant elevation in malondialdehyde (MDA) content and ultimately causing oxidative damage to cell membranes [60]. The MDA content in the roots of *Rht-D1b* decreased significantly (Figure 3D), which may be attributed to efficient ROS scavenging that enables this line to maintain superior membrane stability. By contrast, there was no significant change in MDA content in the shoot and root tissues of *Rht-D1a* under salt stress (Figure 3C), which may be attributable to the impaired function of its antioxidant defense system, leading to decreased membrane stability. Intracellular antioxidant enzymes can effectively scavenge excess accumulated ROS under stress conditions, thereby protecting cells from oxidative damage [61].

As the first line of defense in the antioxidant system, SOD is responsible for rapidly converting superoxide anions into hydrogen peroxide and oxygen [62]. Salt stress induced a significant increase in SOD activity in the shoots of both *Rht-D1a* and *Rht-D1b* (Figure 3E), indicating that both allelic types can perceive salt stress signals and activate the SOD-centered enzymatic antioxidant defense system. Under salt stress, SOD activity in the roots of *Rht-D1b* decreased significantly, while no significant difference was detected in *Rht-D1a* (Figure 3F). POD and CAT can decompose hydrogen peroxide produced via SOD-catalyzed reaction into H_2_O and O_2_, thereby scavenging ROS, alleviating membrane lipid peroxidation damage, and maintaining cellular redox homeostasis [63]. Under salt stress, POD activity in the shoots of *Rht-D1a* decreased significantly, whereas that of *Rht-D1b* increased significantly (Figure 3G); by contrast, CAT activity in both *Rht-D1a* and *Rht-D1b* showed no significant change (Figure 3I). This indicates that in the shoots of *Rht-D1a*, the reduction in POD activity coupled with the absence of a CAT response leads to net accumulation of hydrogen peroxide generated via SOD catalysis, ultimately resulting in aggravated oxidative stress. Although salt stress induced a significant increase in POD activity in the roots of *Rht-D1a* (Figure 3H), it caused a significant decrease in CAT activity (Figure 3J); in contrast, both POD and CAT activities were significantly increased in the roots of plants carrying the *Rht-D1b* allele, indicating that the *Rht-D1b* line might possess a stronger hydrogen peroxide scavenging capacity through the synergistic upregulation of POD and CAT in root tissues. Furthermore, the roots of *Rht-D1b* can lower hydrogen peroxide production by downregulating SOD activity, ultimately driving an optimized shift in the plant antioxidant defense strategy.

### 3.3. Transcriptome Quality Assessment, Dynamic DEG Analysis and Functional Enrichment Under Salt Stress

**Figure 4:**
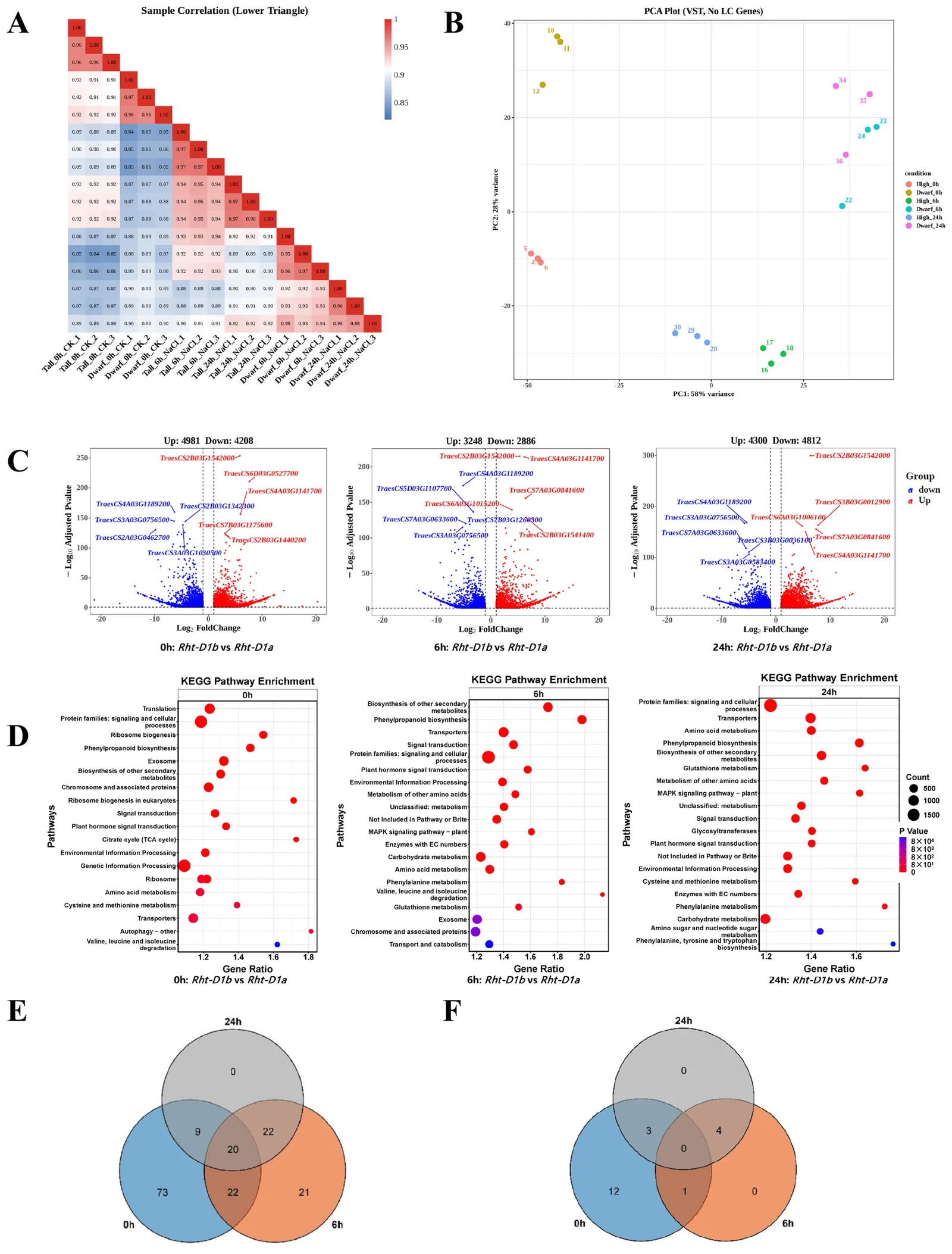
Transcriptome data quality evaluation and DEG enrichment analysis of tall and dwarf wheat under salt stress. (**A**) Sample correlation heatmap of wheat root transcriptome samples; (**B**) principal component analysis (PCA) of wheat root transcriptome samples; (**C**) volcano plots of DEGs between tall and dwarf wheat genotypes at 0 h, 6 h and 24 h under 200 mmol/L NaCl salt stress; (**D**) KEGG pathway enrichment analysis of DEGs at three salt stress time points; (**E**) Venn diagram analysis of the upregulated genes in the *POD* family across three time points; and (**F**) Venn diagram analysis of the upregulated genes in the *HKT*, *TaNHX*, *SOS*, *TaCNGC*, *TaRboh*, *SOD* and *TaCAT* family across three time points.

Transcriptome data quality of *Rht-D1a* (tall) and *Rht-D1b* (semi-dwarf) wheat roots under 200 mmol/L NaCl stress was validated via correlation analysis and PCA (Figure 4A,B). High correlations among biological replicates confirmed reliable sequencing data. PCA showed that PC1 and PC2 explained 58% and 28% of total transcriptomic variation (86% cumulative), with samples clearly separated by genotype and salt treatment duration, indicating both factors substantially modulated wheat root transcriptomes.

Dynamic transcriptomic changes between the two genotypes were analyzed at 0 h, 6 h, and 24 h of salt stress (Figure 4C). In total, 9189 DEGs (4981 upregulated, 4208 down-regulated) were identified at 0 h, revealing prominent basal transcriptomic differences and distinct inherent physiological characteristics between the two genotypes (Table S2). The number of DEGs decreased to 6134 (3248 upregulated, 2886 down-regulated) at 6 h (a 33.3% reduction compared with 0 h) and rebounded to 9112 (4300 upregulated, 4812 down-regulated) at 24 h (a 48.5% increase compared with 6 h), showing a dynamic trend of initial decline followed by elevation (Tables S3 and S4). The reduced DEG number at 6 h suggested convergent early transcriptional responses, while the DEG rebound and predominance of down-regulated genes at 24 h indicated aggravated transcriptomic divergence and genotype-specific long-term salt adaptation strategies in *Rht-D1b*.

KEGG enrichment analysis of DEGs across three time points revealed conserved pathways including phenylpropanoid biosynthesis, plant hormone signal transduction, substance transport, and secondary metabolism (Figure 4D), suggesting core genotypic differences in stress signal perception and metabolic processes. Time-specific functional enrichment was observed in DEGs. Basal DEGs at 0 h were enriched in basal metabolism and genetic processing pathways, confirming intrinsic genotypic differences. At 6 h, DEGs were mainly enriched in signal transduction, antioxidant defense, secondary metabolism and substance-transport pathways, reflecting divergent early stress response efficiency. At 24 h, DEGs were further enriched in secondary metabolism, plant MAPK signaling and antioxidant pathways, indicating a shift toward metabolic remodeling and cellular homeostasis maintenance for long-term salt adaptation.

Enrichment analysis of upregulated DEGs combined with physiological data suggested a staged transcriptional response pattern in the *Rht-D1b* line. The upregulated basal metabolism before stress might reflect a possible pre-defense or pre-adaptive state. Early salt stress was associated with rapid signal transduction, antioxidant defense and ion transport. Prolonged stress induced enhanced carbon metabolism, peroxisome activity and antioxidant biosynthesis, which could help to reduce ROS and MDA accumulation, stabilize cell membranes, and maintain ion homeostasis. In conclusion, the presence of *Rht-D1b* might contribute to enhancing salt tolerance via stage-specific transcriptional regulation, including efficient stress signal transduction, effective oxidative damage alleviation, and stable ion homeostasis, which distinguishes it from *Rht-D1a* under salt stress.

### 3.4. Analysis of Differential Expression of Salt-Tolerance-Related Gene Families Between Two Genotypes

To further dissect the core gene families driving the divergent salt-responsive pathways uncovered by KEGG enrichment and reveal the molecular mechanism responsible for the superior salt tolerance of semi-dwarf *Rht-D1b* relative to tall *Rht-D1a*, we systematically characterized the temporal expression patterns of eight well-documented salt-tolerance-related gene families linked to ion transport and ROS scavenging and identified a total of 938 genes belonging to these eight families across the wheat reference genome (Table S5). These genes were divided into two functional modules: ion transport and homeostasis (*HKT*, *TaNHX*, *SOS*, *TaCNGC*), and ROS scavenging and antioxidant defense (*TaRboh*, *SOD*, *TaCAT*, *POD*). Interestingly, the *POD* family contained the largest number of identified genes (650), among which 167 were differentially expressed genes (DEGs) (Table S5). This large number is likely associated with the hexaploid nature of wheat and frequent duplication events within this family. The other seven families contributed only 288 DEGs, accounting for a relatively small proportion. This indicated that the *POD* family represented the most transcriptionally responsive component during salt stress. Based on the TPM expression data in Table S5, we compared only the upregulated DEGs across the three time points (0, 6, and 24 h), separately in the *POD* family and in the other seven gene families.

According to the Venn diagram analysis of the upregulated genes in the *POD* family (Figure 4E), a total of 20 DEGs were upregulated in expression across all time points. At 6 h of salt stress, 85 DEGs were transiently upregulated. Among them, 22 genes were upregulated at both 0 h and 6 h. At 24 h of salt stress, 51 DEGs were upregulated. Of these, nine genes were upregulated at both 0 h and 24 h, while 22 genes were commonly upregulated at 6 and 24 h. Interestingly, no *POD* family genes were identified as being specifically upregulated only at 24 h. Overall, the results point to the *POD* family exhibiting consistently higher expression in the *Rht-D1b* line than in the *Rht-D1a* line at 0, 6, and 24 h, together with additional early-induced expression after salt treatment. As a major ROS-scavenging enzyme family, *POD* genes may be rapidly and continuously activated at the transcriptional level in the *Rht-D1b* line. This pattern is consistent with a possible primed antioxidant state and may also reflect basal expression differences between the two lines.

For the other seven gene families, including *HKT*, *TaNHX*, *SOS*, *TaCNGC*, *TaRboh*, *SOD*, and *TaCAT*, the Venn diagram analysis showed that no DEGs were upregulated in expression across all the time points (Figure 4F). At 0 h, 16 DEGs were detected in the dwarf line *Rht-D1b*. These belonged to five families: *HKT* (1), *TaNHX* (1), *SOS* (9), *TaCNGC* (2), and *TaRboh* (2). This indicated that the basal differences were mainly related to genes involved in ion transport, ion homeostasis, and ROS-related responses (Table S6). At 6 h of salt stress, five DEGs were upregulated, which were derived from the *HKT* (1), *TaNHX* (1), *TaCNGC* (1), and *TaRboh* (2) families (Table S7). Among them, *TraesCS1D03G0763300* (from *TaCNGC*) was upregulated at both 0 h and 6 h. At 24 h of salt stress, seven DEGs were upregulated, belonging to the *HKT* (2), *TaNHX* (2), *SOS* (1), and *TaRboh* (2) families (Table S8). Three of these genes were upregulated at both 0 h and 24 h: *TraesCS7B03G0854300* (*HKT*), *TraesCS2D03G0258000* (*TaNHX*), and *TraesCS1B03G0759300* (*SOS*). The other four genes were upregulated only at 6 h and 24 h: *TraesCS2D03G0965500* (*HKT*), *TraesCS2A03G0249200* (*TaNHX*), *TraesCS1D03G0682400* (*TaRboh*), and *TraesCS1D03G0682800* (*TaRboh*). Overall, these ion transport-related gene families showed a sequential activation pattern during salt stress. This pattern suggests that *Rht-D1b* might help to maintain ion homeostasis through *HKT*-mediated Na^+^ retrieval, *TaNHX*-mediated vacuolar Na^+^ sequestration, *SOS*-mediated Na^+^ extrusion, and *TaCNGC*-mediated Ca^2+^ signaling. Meanwhile, *TaRboh* mediates ROS production, *SOD* converts superoxide (O_2_^−^) into H_2_O_2_, and *TaCAT* catalyzes H_2_O_2_ decomposition. Together, these gene families coordinate ion homeostasis, ROS signaling, and cellular redox homeostasis, providing a molecular basis for the stronger salt tolerance of *Rht-D1b*.

## 4. Discussion

This study examined the regulatory effects of the wheat Green Revolution dwarfing gene *Rht-D1b* on salt stress tolerance. Through combined phenotypic, physiological, and transcriptomic analyses, it was demonstrated that *Rht-D1b* may contribute to enhancing salt tolerance in wheat. Notably, this enhancement is not attributed to the dwarfing effect alone but rather to the synergistic interaction of multi-level physiological and transcriptional reprogramming. These findings broaden our understanding of the functional roles of Green Revolution genes in abiotic stress adaptation and provide new insights into the theoretical framework of wheat salt tolerance. They also offer valuable genetic resources and a theoretical foundation for the molecular breeding of salt-tolerant, high-yielding wheat varieties. The mechanisms underlying these effects will be discussed in detail in the following sections, focusing on two key perspectives: resource allocation and transcriptional pre-adaptation, as well as ion transport and antioxidant defense.

### 4.1. Rht-D1b Is Implicated in Salt Tolerance Through Resource Reallocation and Transcriptional Pre-Adaptation

Dwarfing alleles (reduced height, *Rht*) are an important breeding tool for increasing wheat grain yields [64]. *Rht-D1b* is a classic semi-dwarfing allele of the Green Revolution in wheat [65]. Its functions in reducing the response to gibberellin (GA) signaling, inhibiting stem elongation, strengthening lodging resistance, and boosting yield potential under high-density planting have been well characterized [66,67,68]. Previous studies reported that semi-dwarf wheat lines exhibit altered carbon-nitrogen allocation patterns [69]. The *Rht* alleles affect not only plant height but also traits related to resource use efficiency, such as specific leaf area, mean residence time of nitrogen, grain number on spike, and harvest index [13]. In addition, population multi-omics analyses demonstrate that *Rht-D1b* enlarges root meristem size and synchronously increases the length and width of cells in mature root zones, which significantly raises total root length, root surface area and root volume and further improves the root-to-shoot ratio [70].

This study found that under 200 mM NaCl stress, the genotype carrying *Rht-D1b* maintained growth better than the wild-type *Rht-D1a*. Specifically, it showed a smaller reduction in plant height, less inhibition of shoot fresh and dry weights, and no significant decline in root fresh weight. This indicates that the advantage of *Rht-D1b* is not merely a passive growth reduction caused by dwarfing. Instead, it is more likely associated with active adjustments of growth processes and resource allocation under stress. Therefore, the stronger growth maintenance of *Rht-D1b* under salt stress may arise from moderately restricting shoot elongation and reducing assimilate competition from rapid stem expansion. This would allocate more resources to root development, osmotic adjustment, and ion homeostasis. This explanation is consistent with the results of this study, which showed that *Rht-D1b* and *Rht-D1a* already exhibited pronounced transcriptomic differences before salt stress (0 h) (Figure 4C). Similarly, research has reported numerous differentially expressed genes in root tissues of salt-tolerant and salt-sensitive wheat lines prior to stress exposure [71]. Tolerant genotypes maintain higher basal levels of stress-related transcripts, a pattern that appears conserved across species [72]. The basal transcriptomic differences observed at 0 h could be attributed to *Rht-D1* allelic variation, residual genetic-background polymorphisms, or a combination of both. If these differences are driven by *Rht-D1b*, they might confer a pre-adaptive state that could enhance the plant’s ability to respond to subsequent salt stress.

At the molecular level, the advantage of *Rht-D1b* is also closely related to the integration of stress signals mediated by the DELLA protein. The *Rht-D1* gene encodes DELLA proteins, which are negative regulators in the GA signaling pathway. DELLAs not only mediate height reduction by repressing GA responses but also act as key hubs for integrating environmental stress signals [73]. Previous studies have shown that DELLA accumulation can promote plant stress tolerance by reducing reactive oxygen species accumulation and enhancing the expression of ROS-detoxification enzyme-related genes [74]. This provides important support for the potential involvement of *Rht-D1b* in salt adaptation. The activation of these integrative functions of coercive signals may have further driven the downstream physiological phenotypes observed in this study. In the study, *Rht-D1b* displayed significantly elevated SPAD values and more stable biomass under salt treatment (Figure 1). This demonstrates that it maintained better chlorophyll status and photosynthetic function under stress, thereby supplying essential carbon skeletons and energy for stress responses. This observation aligns with reports that salt-tolerant wheat genotypes retain higher chlorophyll and carotenoid contents under NaCl stress [75]. In addition, *Rht-D1b* accumulated less Na^+^ in shoots, maintained a higher K^+^/Na^+^ ratio, showed lower ROS and MDA accumulation, and exhibited stronger POD and CAT activities in roots (Figure 3). These data highlight that the salt tolerance advantage of *Rht-D1b* also involves the maintenance of ion homeostasis and mitigation of oxidative damage.

In summary, this study suggests that the superior performance of *Rht-D1b* under salt stress is likely due to synergistic effects of multiple factors. These include dwarfing regulation, optimized resource allocation, basal transcriptomic differences, photosynthetic maintenance, ion homeostasis regulation, and enhanced antioxidant defense, rather than a single effect of reduced plant height. Therefore, *Rht-D1b* is not only an important allele for improving plant architecture and lodging resistance in wheat. It can also serve as a genetic resource that combines an ideal plant type with salt tolerance potential. It holds considerable value for wheat improvement in saline-alkali lands and for the development of salt-tolerant semi-dwarf germplasm.

### 4.2. Ion Transport and ROS Scavenging Are Associated with Enhanced Salt Tolerance in Rht-D1b

The salt-tolerance advantage of *Rht-D1b* may result from the joint regulation of antioxidant defense, Na^+^ homeostasis, and Ca^2+^-ROS signaling. The *POD* genes identified in the wheat genome represent the largest family among the eight salt-tolerance-related gene families. This large family size is not unexpected—the class III POD gene family is inherently large in plants, participating in diverse physiological processes, including cell wall loosening, lignification, and ROS scavenging, and has been reported to undergo frequent duplication events in various species [76,77,78]. In wheat, the hexaploid nature (A, B, and D subgenomes) further triplicates the genomic repertoire of each gene family. More importantly, a total of 396 duplication events, primarily through segmental and tandem duplications, have been identified within the *TaPOD* family during wheat evolution [79]. The other families, despite also having three subgenomic copies, lack such extensive duplication-driven expansion and are therefore much smaller in member count. This size disparity likely gives the *POD* family a larger pool of stress-responsive candidates, consistent with its predominant transcriptional activation under salt stress. Compared with other salt-tolerance-related gene families, the *POD* family acts as the core antioxidant module in *Rht-D1b*. It shows a more persistent transcriptional response to salinity. This pattern is consistent with both a primed antioxidant state in the *Rht-D1b* line before stress and rapid activation of ROS scavenging upon salt exposure. This transcriptional pattern is consistent with the physiological data: under salt stress, *Rht-D1b* exhibited significantly lower ROS and MDA levels than *Rht-D1a*, together with markedly increased POD and CAT activities. These results indicate that the sustained activation of POD-related genes may reduce membrane lipid peroxidation through enhanced H_2_O_2_ clearance. The ROS signal detected by DCFH-DA in this study primarily reflects H_2_O_2_ and hydroxyl radicals (·OH). Elevated SOD activity drives the dismutation of O_2_^−^ to H_2_O_2_, so the increased ROS signal most likely represents H_2_O_2_ accumulation. H_2_O_2_ is a relatively mild oxidant that does not directly attack membrane lipids; lipid peroxidation requires ·OH, generated from H_2_O_2_ via the Fenton reaction in the presence of free transition metal ions such as Fe^2+^ or Cu^2+^ [80]. In plant cells, ferritin sequesters free iron in a biologically unavailable form, and salt stress can further induce ferritin accumulation, thereby exacerbating the scarcity of labile iron [81]. The ascorbate-glutathione cycle (APX) and non-enzymatic antioxidants such as ascorbate and glutathione also participate in H_2_O_2_ scavenging, though these were not measured in the present study. Therefore, elevated H_2_O_2_ does not necessarily translate into increased MDA levels—MDA accumulation depends not only on ROS production but also on the efficiency of ·OH generation and the robustness of membrane protection mechanisms. Measuring ferritin expression and APX activity in future experiments would help clarify the full redox network operating in the two *Rht-D1* lines. Previous studies have shown that overexpression of the wheat class III peroxidase gene *TaPRX-2A* increased SOD, POD, and CAT activities and decreased ROS and MDA accumulation in transgenic wheat. In addition, *TaPRX-2A* enhanced salt tolerance by upregulating stress-related genes, including *RD22*, *FeSOD*, and *CAT* [82]. Therefore, the sustained response of POD genes may be an important reason why *Rht-D1b* maintains lower ROS levels and stronger membrane stability under salt stress.

Genes from the *HKT*, *TaNHX*, and *SOS* families may regulate Na^+^ homeostasis in *Rht-D1b* through a multi-tiered network. *HKT*-related genes are involved in long-distance Na^+^ transport and limit Na^+^ accumulation in shoots [83]. In rice, salt stress induces *OsHKT1;1* expression, which helps control Na^+^ accumulation in leaves and reduces Na^+^ toxicity [84]. Suppression of *OsHKT2;1* can reduce Na^+^ uptake at specific stages of salt exposure [85]. In Arabidopsis, *AtHKT1;1* promotes Na^+^ unloading from xylem vessels, excluding Na^+^ from shoots [86]. These findings about homologs in rice and Arabidopsis support the role of *HKT* genes in Na^+^ homeostasis and salt tolerance. The *TaNHX* family may reduce cytosolic Na^+^ toxicity through vacuolar sequestration [87]. In rice, salt stress markedly induces *OsNHX* gene expression, and *OsNHX1*, *OsNHX2*, *OsNHX3*, and *OsNHX5* are suggested to participate in the vacuolar compartmentalization of excess cytosolic Na^+^ and K^+^ [88]. Overexpression of the wheat vacuolar Na^+^/H^+^ antiporter TNHX1 and H^+^-pyrophosphatase TVP1 enhanced salt-stress tolerance in transgenic Arabidopsis, supporting the role of wheat NHX-type antiporters in vacuolar ion sequestration and salt-tolerance improvement [89]. The *SOS* family regulates Na^+^ efflux through the Ca^2+^-dependent CBL–CIPK/SOS module [90]. The OsCBL4/OsSOS3–OsCIPK24/OsSOS2–OsSOS1 module regulates Na^+^ homeostasis in rice, and reverse-genetic evidence shows that SOS1-mediated Na^+^ flux is essential for salt tolerance [91]. In Arabidopsis, *AtCBL9* loss-of-function mutants show a salt-sensitive phenotype under salt stress, with elevated Na^+^ content, cytosolic Ca^2+^, and ROS levels in root tips [92]. Given the evolutionary conservation of the SOS pathway, its homologous genes in *Rht-D1b* may contribute to salt tolerance through Ca^2+^-dependent Na^+^ efflux. Collectively, *Rht-D1b* might not rely on a single Na^+^ transport route. Instead, it may reduce ion toxicity through *HKT*-mediated Na^+^ transport regulation, *TaNHX*-mediated vacuolar Na^+^ sequestration, and *SOS*-mediated Na^+^ efflux. *TaCNGC* and *TaRboh* family members may further integrate Ca^2+^ and ROS signaling [50,93]. *TaCNGC*s may act as Ca^2+^ signal input components and transmit salt-stress signals to downstream defense responses. Studies on chilling stress in rice showed that *OsCNGC9* mediates Ca^2+^ influx, corroborating the essential roles of *CNGC* genes in Ca^2+^ signaling and responses to abiotic stresses [94]. The *Rboh* family participates in salt-stress responses by regulating ROS production and signal transmission. In rice, *OsRbohB* is significantly induced by salinity and contributes to ROS production, whereas *OsRbohH* is directly targeted by the transcription factor *OsERF2* and negatively regulates salt tolerance [95,96]. Wheat homologs (*TaRboh*s) may similarly modulate ROS production and signaling during salt exposure. Ca^2+^ triggers ROS production by directly activating RBOH proteins via EF-hand Ca^2+^-binding domains or indirectly through Ca^2+^-dependent phosphorylation [97]. Thus, *Rbohs* mediate Ca^2+^-dependent ROS production. Wheat lines carrying *Rht-D1b* (encoding an altered-function DELLA protein) differ markedly from wild-type lines in the accumulation of major cations, including Ca^2+^, Na^+^, and K^+^ [98]. Salt stress in *Rht-D1b* lines likely triggers Ca^2+^ influx through *TaCNGC*s. This influx then activates *TaRboh*s to produce ROS via Ca^2+^ binding to EF-hand motifs and Ca^2+^-dependent phosphorylation. The elevated Ca^2+^ and ROS signals may together form an upstream framework that primes the downstream antioxidant system, consistent with the enhanced POD and CAT activities observed in *Rht-D1b*. Within this signaling framework, the reduced SOD activity in *Rht-D1b* roots does not necessarily indicate weaker antioxidant capacity. Rather, this change may suggest that fewer superoxide anions were converted into H_2_O_2_, while the increased activities of POD and CAT helped remove H_2_O_2_ more effectively. Overall, the salt tolerance of *Rht-D1b* may be associated with a combined regulatory network. In this network, *POD* genes provide sustained antioxidant defense. *HKT*, *TaNHX*, and *SOS* genes work together to reduce Na^+^ toxicity; *TaCNGC* and *TaRboh* link Ca^2+^ signaling with ROS signaling; and *SOD*, *TaCAT*, and *POD* jointly regulate ROS transformation and clearance. These coordinated responses may explain why *Rht-D1b* shows better ion balance, lower oxidative damage, and stronger stress tolerance than *Rht-D1a*.

## 5. Conclusions

Taken together, our results suggest that the semi-dwarf allele *Rht-D1b* contributes to enhanced salt tolerance in wheat through coordinated physiological responses and early transcriptional activation. Compared with *Rht-D1a*, wheat carrying *Rht-D1b* maintained better ion homeostasis and redox balance under salt stress, resulting in improved salt tolerance. Consistent with these physiological responses, multiple gene families involved in Na^+^ transport, vacuolar sequestration, and ROS scavenging exhibited coordinated transcriptional regulation, linking *Rht-D1b* with salt-stress adaptation. These findings might expand the functional role of *Rht-D1b* beyond plant height regulation and provide new insights into the contribution of Green Revolution genes to abiotic stress tolerance. The candidate genes identified in this study provide valuable genetic resources for future molecular breeding. Further functional characterization is required to determine their specific roles in salt-stress adaptation.

A limitation of this study is that untreated controls were not collected at 6 and 24 h. Therefore, the 0 h samples served as the baseline, and the DEGs identified at 6 and 24 h represent changes relative to this baseline rather than solely a direct response to salt stress. Since these two lines are not strictly near-isogenic and exhibit significant transcriptomic differences at 0 h, these DEGs may reflect both basal expression differences and a component associated with the response to salt stress. The observed differences cannot be exclusively attributed to a salt-stress response mediated by *Rht-D1b*. Accordingly, the specific contribution of *Rht-D1b* to salt tolerance remains to be confirmed using independent near-isogenic lines or CRISPR-edited materials with time-matched controls.

## Data Availability

The datasets used in the current study are available from the corresponding author upon reasonable request. RNA-Seq data have been deposited in the NCBI BioProject accession number PRJNA1499890.

## Supplementary Materials

The following supporting information can be downloaded at https://www.mdpi.com/article/10.3390/life16101632/s1. Table S1: Genome-wide SNP distribution and polymorphism statistics between Zhongyou 9507 (Rht-D1a) and Zhongyou 9507s (Rht-D1b) based on 50K SNP chip genotyping; Table S2: Differentially expressed genes between Rht-D1a tall and Rht-D1b dwarf near-isogenic lines under 0 h NaCl treatment in seedling stage; Table S3: Differentially expressed genes between Rht-D1a tall and Rht-D1b dwarf near-isogenic lines under 6 h NaCl treatment in seedling stage; Table S4: Differentially expressed genes between Rht-D1a tall and Rht-D1b dwarf near-isogenic lines under 24 h NaCl treatment in seedling stage; Table S5: TPM values of eight salt-responsive gene families under 0, 6, and 24 h NaCl treatment in Rht-D1 near-isogenic lines; Table S6: Differentially expressed salt-responsive gene families in Rht-D1 NILs after 0 h of NaCl stress; Table S7: Differentially expressed salt-responsive gene families in Rht-D1 NILs after 6 h of NaCl stress; Table S8: Differentially expressed salt-responsive gene families in Rht-D1 NILs after 24 h of NaCl stress.

## Abbreviations

CAT	Catalase
CBL	Calcineurin B-like Protein
CIPK	CBL-interacting Protein Kinase
CNGC	Cyclic Nucleotide-Gated Channel
DEG	Differentially Expressed Gene
GA	Gibberellin
HKT	High-Affinity Potassium Transporter
KEGG	Kyoto Encyclopedia of Genes and Genomes
MDA	Malondialdehyde
NHX	Na^+^/H^+^ Exchanger
NILs	Near-isogenic Lines
PCA	Principal Component Analysis
POD	Peroxidase
Rboh	Respiratory Burst Oxidase Homolog
RNA-seq	RNA Sequencing
ROS	Reactive Oxygen Species
SOD	Superoxide Dismutase
SPAD	Soil–Plant Analysis Development
TPM	Transcripts Per Million
